# Transdiagnostic markers across the psychosis continuum: a systematic review and meta-analysis of resting state fMRI studies

**DOI:** 10.3389/fpsyt.2024.1378439

**Published:** 2024-06-04

**Authors:** Giuseppe Pierpaolo Merola, Livio Tarchi, Luigi F. Saccaro, Farnaz Delavari, Camille Piguet, Dimitri Van De Ville, Giovanni Castellini, Valdo Ricca

**Affiliations:** ^1^ Psychiatry Unit, Department of Health Sciences, University of Florence, Florence, Italy; ^2^ Psychiatry Department, Geneva University Hospital and Faculty of Medicine, Geneva University Hospital, Geneva, Switzerland; ^3^ Neuro-X Institute, École Polytechnique Fédérale de Lausanne, Geneva, Switzerland; ^4^ Developmental Imaging and Psychopathology Laboratory, University of Geneva School of Medicine, Geneva, Switzerland; ^5^ General Pediatric Division, Geneva University Hospital, Geneva, Switzerland; ^6^ Department of Radiology and Medical Informatics, University of Geneva, Geneva, Switzerland

**Keywords:** schizophrenia, schizoaffective disorder, bipolar disorder, ALFF (amplitude of low frequency fluctuation), fALFF (fractional amplitude of low frequency fluctuations)

## Abstract

Psychotic symptoms are among the most debilitating and challenging presentations of severe psychiatric diseases, such as schizophrenia, schizoaffective, and bipolar disorder. A pathophysiological understanding of intrinsic brain activity underlying psychosis is crucial to improve diagnosis and treatment. While a potential continuum along the psychotic spectrum has been recently described in neuroimaging studies, especially for what concerns absolute and relative amplitude of low-frequency fluctuations (ALFF and fALFF), these efforts have given heterogeneous results. A transdiagnostic meta-analysis of ALFF/fALFF in patients with psychosis compared to healthy controls is currently lacking. Therefore, in this pre-registered systematic review and meta-analysis PubMed, Scopus, and Embase were searched for articles comparing ALFF/fALFF between psychotic patients and healthy controls. A quantitative synthesis of differences in (f)ALFF between patients along the psychotic spectrum and healthy controls was performed with Seed-based d Mapping, adjusting for age, sex, duration of illness, clinical severity. All results were corrected for multiple comparisons by Family-Wise Error rates. While lower ALFF and fALFF were detected in patients with psychosis in comparison to controls, no specific finding survived correction for multiple comparisons. Lack of this correction might explain the discordant findings highlighted in previous literature. Other potential explanations include methodological issues, such as the lack of standardization in pre-processing or analytical procedures among studies. Future research on ALFF/fALFF differences for patients with psychosis should prioritize the replicability of individual studies.

**Systematic review registration:**

https://osf.io/, identifier (ycqpz).

## Introduction

Bipolar disorders, Schizophrenia and Schizoaffective Disorder are serious, debilitating, and prevalent psychiatric disorders that have been traditionally considered separate entities. However, recent research suggests that they may exist on a continuous spectrum rather than being distinct disorders (1). This idea is supported by the overlapping symptoms, genetic risk factors, and neural mechanisms that have been found between these two conditions (2, 3). As recently proved by a recent large GWAS meta-analysis (4), the genetic correlation between schizophrenia and bipolar disorder of both types I and II is the highest across most psychiatric disorders (~0.7). Additionally, studies have shown that individuals diagnosed with one of these disorders are often found to have subthreshold symptoms of the other (5). This has led to a growing recognition that the traditional categorical approach to mental health diagnosis may not accurately capture the complexity of psychiatric conditions and that a dimensional approach may be more appropriate.

Both Schizophrenia and Bipolar Disorder have been hypothesized to be influenced by neurodevelopmental processes, possibly occurring during adolescence (6). Nonetheless, the precise etiological link between neurodevelopment and psychosis has not yet been clarified, while preliminary experimental evidence has focused on the role of intrinsic brain activity as conferring a special risk and informing clinical severity and prognosis (7–9).

Neuroimaging studies have traditionally investigated intrinsic brain activity according to two diverging fields of interests: the characterization of functional connectivity between brain regions, and the spatiotemporal nature of its signal (10). Spatiotemporal brain activity has been operatively explored mainly through two measures, namely the absolute and relative amplitude of low-frequency fluctuations (ALFF and fALFF, respectively). While ALFF focuses on an absolute quantity of power over a frequency spectrum, fALFF assesses the relative contribution of low-frequencies to the whole.

The ALLF methods hold significance in psychiatric disorders compared to functional connectivity analysis due to its utilization of low-frequency fluctuations in the BOLD signal, which reflect spontaneous neural activity. Unlike connectivity analysis, this approach is not restricted to individual regions as no single brain area has been conclusively postulated to be involved in psychopathology of these disorders. Therefore, the exploration of low-frequency fluctuations patterns may provide a more informative approach with potential clinical implications for psychiatric disorders, while also being easily leveraged by meta-analytic analyses. Nonetheless, while more easily amenable to meta-analytical techniques, the biological interpretation of ALFF and fALFF measures in neuroimaging studies remains unclear, and the potential for translational approaches thus remain limited.

A previous meta-analysis, on Schizophrenia alone, showed reduced ALFF and fALFF bilaterally in the postcentral gyrus, bilaterally in the precuneus, in the left inferior parietal gyri and in the right occipital lobe at rest as compared to controls (9). Moreover, the same-meta-analysis showed increased ALFF in the right putamen, right inferior frontal gyrus, left inferior temporal gyrus and right anterior cingulate cortex. Similar evidence has been gathered for patients with Bipolar Disorder (8), where patients exhibited increased ALFF in the ventral prefrontal cortex, dorsolateral prefrontal cortex, mid occipital lobe, insular cortex, and putamen, as well as decreased ALFF in the medial occipitotemporal gyrus. The extent to which the observed similarities in changes among individuals with bipolar disorder and those with schizophrenia can be conceptualized within a spectrum of continuum alongside other psychotic disorders remains uncertain. Nonetheless, no previous meta-analytical work assessed transdiagnostic markers of psychosis throughout the diagnostic continuum of schizophrenia, bipolar disorder, schizoaffective disorder and schizotypal personality disorder.

However, the continuum in psychosis has been the interest of the current literature on the topic. In fact, Lencer et al. found reduced ALFF in patients with psychosis compared to controls bilaterally in the frontal eye field regions, in the supplementary eye fields, bilaterally in the thalamus, in the left orbitofrontal gyrus and in the left superior temporal gyrus (11).

Individual characteristics could also influence the heterogeneity between findings in schizophrenia, bipolar and schizoaffective disorder. In fact, age, sex and clinical severity have been previously observed as potential confounders on cognitive and clinical correlates in neuroimaging studies for these disorders (9; S. 12). Moreover, smoking status has been described as a confounder for the BOLD response in fMRI, potentially masking significant differences in case-control studies (13), while being a common addiction in patients suffering from psychosis.

Therefore, an update in the literature seems timely. Meta-analytical approaches allow neuroimaging studies to improve in the reliability and replicability of their findings (9, 14), while acknowledging the specific limitations of the field, such as low effect sizes, high inter- and intraindividual variability, and considerable constraints to signal-to-noise ratios (15, 16). However, special criticism was reserved for the high heterogeneity of these conclusions (17). Such heterogeneity in psychiatry has been associated with various possible factors, and in particular the weak neurobiological basis on which clinical diagnoses are formulated. Here we employed a meta-analysis exploring whether individual diagnoses along the psychotic spectrum exhibit spatiotemporal divergences, and whether an overall trend can be described. Finally, the role of individual characteristics on these results would be addressed.

### Aims

The primary objective was to quantitatively synthesize the current literature comparing ALFF and fALFF between healthy controls and patients with psychosis, firstly exploring the potential differences across single diagnoses, then comparing the spectrum encompassing Schizophrenia, Schizoaffective and Bipolar Disorder. In order to achieve this, novel methods informed by the risk of inflating Type I statistical errors were employed (18). The secondary objective of the current study was to explore the role of potential confounders such as age, sex, clinical severity, smoking status and duration of illness as influencing current results.

## Methods

### Search strategy and selection criteria

This pre-registered, systematic review and meta-analysis was conducted following the Preferred Reporting Items for Systematic Reviews and Meta-analyses (PRISMA) guidelines (19), and was pre-registered in the registries of the Open Science Framework (OSF; https://osf.io/ycqpz).The search algorithm was run in February 2023, in the following databases: PubMed (including MEDLINE, Bookshelf, and part of PMC), Scopus, and Embase. The search strategy included intentionally broad terms:

(fmri OR mri OR “functional MRI”) AND (alff OR falff OR “Amplitude of low frequency fluctuations” OR “BOLD variability”) AND (schizophrenia OR psychosis OR psychoti* OR schizoaff* OR schizo* OR bipol* OR spectrum OR mania OR maniac OR hypoman*).

### Ethics approval and informed consent

All included studies were approved by the local Institutional Research Board (IRB), and fully complied with the Helsinki Declaration for research studies on human participants. Please see included studies in the references for further details on the procedures adopted for informed consent. Ethics approval for the present systematic review and meta-analysis was waived by the local IRB (CEAVC, Florence, Italy), as no human or animal participant was enrolled in the study.

### Data screening

Data screening was performed by three independent researchers (G.P.M, L.T., L.F.S.). Any discrepancy was discussed until a consensus was reached. Initially, duplicate references were manually excluded. The remaining articles were screened by title and abstract, and the full texts identified were further inspected for eligibility against *a priori* defined exclusion and inclusion criteria. As further detailed in **Supplementary Table 1**, we included original articles in English that met the following Participants, Interventions, Comparators, Outcomes, and Study design (PICOS) criteria (detailed in **Supplementary Table 1**); we included all studies that compared psychotic spectrum patients (schizophrenia, bipolar disorder or schizoaffective disorders) to healthy controls. We included all study designs focusing on resting state and using ALFF/fALFF as an outcome apart from case reports, case series, conference abstracts and presentations, reviews, meta-analyses, or systematic reviews. In case a cohort study was included, and an intervention was applied in the original study, only the baseline comparisons before treatments were extracted. In order not to inflate statistical significance, studies involving region-based investigations were excluded.

Moreover, as spatiotemporal measures could be influenced by the range of frequencies analyzed, all included studies were screened for potential differences in bandpass filters applied. All included studies applied a lowpass filter of 0.01Hz and a highpass of 0.08Hz, with two exceptions for studies enrolling patients with Schizophrenia (20, 21), and two studies enrolling patients with bipolar disorder. For schizophrenia, one study applied a lowpass filter of 0.008 Hz and highpass of 0.09Hz (21). Another a lowpass of 0.01 and a highpass of 0.10 (20). The repetition time was 2.30 seconds in the former and 2 seconds in the latter (total scan time 600 seconds/260 timepoints, 21; 420 seconds/210 timepoints, 20). For Bipolar disorder, both studies applied a lowpass filter of 0.01 and highpass of 0.10 (H. 22, 23), with a repetition time of 2 and 2.53 seconds respectively (total scan time 480 seconds/200 timepoints; 600 seconds/240 timepoints). As a Fourier transform was applied to estimate the amplitude of each component in measuring both ALFF and fALFF, the divergent frequency estimation given by the temporal resolution at a repetition time between 2 and 3 seconds was deemed insufficient to cause potential interference on the results. Indeed, the frequency resolution is given by


$$\frac{1}{NT\;*\;TR}$$

where *NT* is the number of timepoints in the series and *TR* is the repetition time (24). The four studies would therefore have a frequency resolution above 0.002 Hz when accounting for volumes discarded due to the transient effects in amplitude observed until the MRI scanner achieves steady state (25).

As outlined in more details in the Data Extraction section, the outcome was the T-values associated with the specific MNI coordinates of the findings (26).

**Figure 1:**
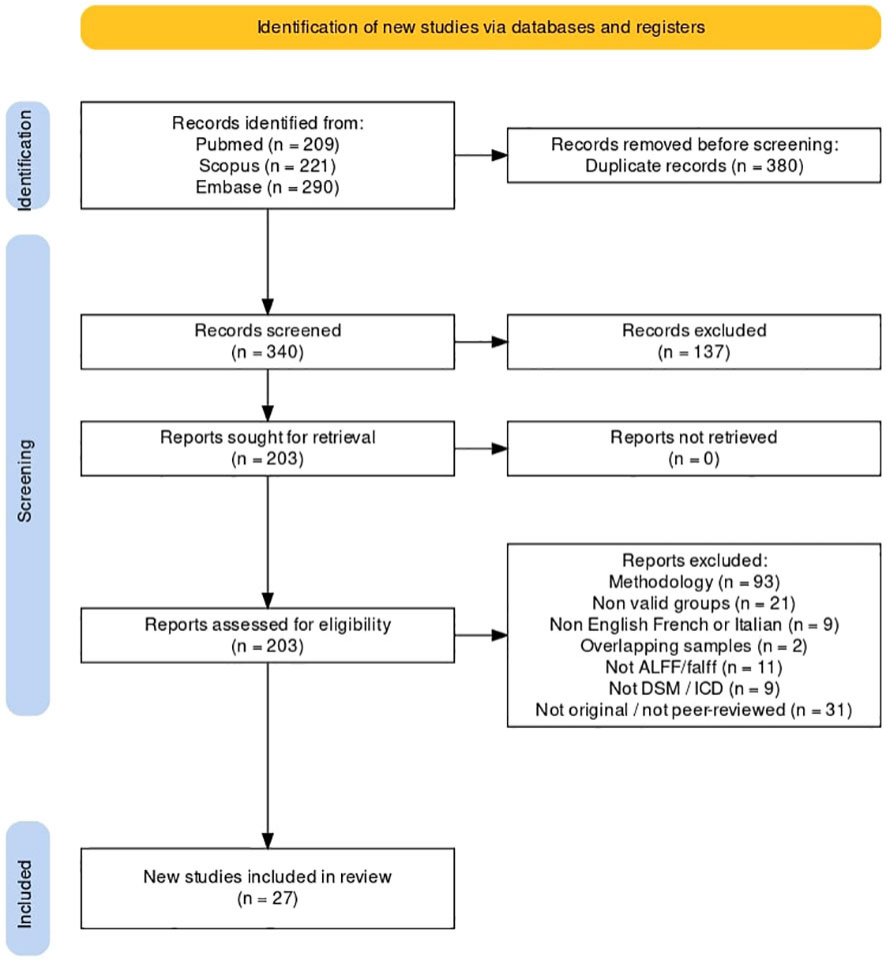
Systematic search flowchart. Description of the searching, screening and data extraction phases according to PRISMA guidelines.

The selection process was documented in the PRISMA flow diagram (**Figure 1**).

### Data extraction

Data extraction was performed by three independent researchers (G.P.M, L.T., L.F.S.). Any discrepancy was discussed until a consensus was reached. The following variables were extracted from each article: authors and year of publication, methodology, main statistical test used, outcome and significant findings between the variables analyzed, diagnosis of inclusion, number of psychiatric patients and controls, percentage of females in the sample, percentage of smokers in the sample, mean age, mean duration of illness, average dose of antipsychotics by Clozapine equivalents, mean Positive and Negative Syndrome Scale (PANSS) score per group, mean FrameWise Displacement per run and group and stereotactic space of reference. If more than one statistical method were employed and results were homogeneous across methods, we reported the main statistical method, if results differed, we reported each method and indicated which method was employed to obtain each result. Authors were also asked to share a statistical map of results, in order to represent results irrespective of preliminary significance thresholding. However, none of the authors responded to this request, and the meta-analysis was conducted reporting peak activations by coordinate and T-value.

### Quality assessment

The quality of the selected studies was assessed with the Newcastle-Ottawa Scale for case-controls studies (27). The NOS is a widely used risk of bias assessment tool (28, 29) consisting of two sections: one for case-report studies and the other for cohort studies. Studies were evaluated using the NOS considering its three domains: patient selection, comparability, and exposure. The risk of bias and concerns regarding applicability were analyzed for each domain (**Supplementary Table 2**).

### Statistical methods

The present meta-analysis approached neuroimaging evidence according to anisotropic kernels as implemented by Seed-based D Mapping (SDM, version 6.22; https://www.sdmproject.com/; 30). To optimally balance false positives and negatives, we used the default SDM kernel size and thresholds (full anisotropy = 1, isotropic full width at half maximum [FWHM] = 20 mm, and voxel = 2 mm). Then, an univariate linear regression analysis was performed employing the following covariates: patients’ age, sex, duration of illness and PANSS scores.

When testing for multiple hypotheses in the same analysis, the risk of Type I statistical error (false positives) increases as a function of the number of tested associations; thus, FWE (family-wise error) correction for multiple comparisons using common permutation tests was subsequently applied and a significance threshold of *p* < .05 was selected, along a minimum cluster extent of 30 voxels (18). Effect sizes of potential group differences in comparison to controls were estimated as per previous methodological contributions on the topic (31). First, each diagnostic subgroup was assessed individually, then the overall psychotic spectrum was explored. An *a priori* threshold of five studies was selected in order to perform the meta-analysis.

The role of individual characteristics was explored, as a potential confounder to overall results. Meta-analytic synthesis of results was repeated including an estimate for previously listed covariates (sex, age, duration of illness, clinical severity, smoking status) by a Generalized Linear Model, both with and without FWE correction. All models included random effects, in order to account for between-study heterogeneity (31–33). As less than five studies reported the percentage of smokers in the sample, the average dose of antipsychotics by Clozapine equivalents, as well as the mean FrameWise Displacement per run, no further analysis on the contribution of these variables was deemed possible. Publication bias was computed for every significant area from the regression analysis through a test for small-study effect. This statistical test detects whether small studies are more likely to report higher effects, potentially increasing publication chances.

## Results

### Demographic and clinical data

Overall, 720 studies were retrieved by the search string. A total of 27 studies were finally included. Twenty studies enrolled patients affected by Schizophrenia, and among these, 11 yielded data values for ALFF (20, 34–43) and 11 for fALFF (21, 36, 41, 44–50), as some studies enrolled multiple populations, or reported both ALFF and fALFF in the same study.

Six studies enrolled patients with Bipolar disorder. Four investigated ALFF (Y. 7, 8, 22, 51), while 2 fALFF (52, 53). One study enrolled both patients with Schizophrenia and Bipolar disorder without discriminating between the two groups, investigating ALFF (11). No study was found enrolling patients with Schizoaffective disorder.

We summarized the screening processes and detailed reasons of exclusion according to PRISMA criteria in a flowchart (**Figure 1**) (54). The psychosis group was composed of 3071 individuals, and the control group of 3485 individuals. Within the psychosis group, the schizophrenia subgroup was composed of 2800 individuals.

The sample was predominantly characterized by young (mean weighted age for schizophrenia equal to 25.79) but chronic patients (average duration of illness for schizophrenia equal to 26.9 months). The sex distribution showed a slight male preponderance. Demographic and clinical data are displayed in more detail in **Table 1**. Several of the variables mentioned in the Methods (average dose of antipsychotics by Clozapine equivalents, mean Framewise displacement, percentage of smokers) section had to be discarded from further analysis, as insufficient data was available (< 5 studies).

**Table 1A:** Demographic and clinical characteristics (ALFF studies).

Study	Diagnosis	N	Females	Age	Duration of illness	PANSS (pos)	PANSS (neg)	N	Female	Age
		(patients)	(patients, proportion)	(patients, years)	(months)			(controls)	(controls, proportion)	(controls, years)
Chen2022	BD	34	0.51	31.88	71.3	/	/	35	0.5	30.6
Cui2016 (auditory hallucinations)	SCZ	15	0.47	22.53	10.2	/	/	19	0.47	23.79
Cui2016 (without auditory hallucinations)	SCZ	17	0.41	21.24	6.51	31.12	25.53	19	0.47	23.79
Lei2015	SCZ	124	0.61	24.47	6.82	15.26	16.58	102	0.51	24.75
Lencer2019	Spectrum	88	0.43	33		15.8	16.1	50	0.61	35
Li2016	SCZ	20	0.7	22.9	6.4	25.1	18.8	16	0.53	22.4
Li2017 (deficit)	SCZ	41	0.44	23.32	20.15	22.15	27.49	42	0.43	23.29
Li2017 (not deficit)	SCZ	42	0.4	22.86	19.83	25.38	16.48	42	0.43	23.29
Liu2016	SCZ	27	0.44	25.44	18.32	21.56	23.15	27	0.33	27.44
Lu2014		18	0.67	15.1	15.6			18	0.67	14.13
Lui2010	SCZ	34	0.62	24.6	7.8	26.9	19.1	34	0.62	25
Ren2013	SCZ	100	0.59	24.3	6.25	25.11	18.84	100	0.59	24.39
Solis2017 (auditory hallucinations)	SCZ	19	0.32	40.05	193	17.89	21.47	20	/	37.75
Solis2017 (without auditory hallucinations)	SCZ	14	0.43	36.43	96	11.43	14.36	20	/	37.75
Xie2021	SCZ	30	0.43	30.3	21.36	19.65	/	33	0.39	32.03
Xu2014	BD	29	0.55	31.38	/	/	/	29	0.38	30.52
Yu2014	SCZ	69	/	31.7	85.2	12.1	13.4	62	/	29.9
Yu2021	SCZ	22	0.5	33.41	15.48	27.18	19.82	60	0.36	32.87
Zhang2021	BD	56	0.38	29.52	/	/	/	71	0.52	30.61
Zheng2016	SCZ	35	0.43	15.5	6.6	20.42	20.91	30	0.57	15.43

**Table 1B:** Demographic and clinical characteristics (fALFF studies).

Study	Diagnosis	N	Females	Age	Duration of illness	PANSS (pos)	PANSS (neg)	N	Female	Age
		(patients)	(patients, proporion)	(patients, years)	(months)			(controls)	(controls, proportion)	(controls, years)
Athanassiou2022	SCZ	62	0.1	33.7	/	/	/	22	0.18	31
Chai2020	BD	26	0.61	22.7	25	/	/	30	0.6	22.5
Gao2022a	SCZ	57	0.35	31	2.52	26	20.68	50	0.46	28.38
Guo2017	SCZ	28	0.35	22.93	24.14	22.68	21.18	40	0.5	23.28
He2012	SCZ	104	0.53	25.36	39.54			104	0.52	26.61
Hu2016	SCZ	42	0.36	24.86	8.38	25.6	18.17	38	0.34	24.76
Huang2010	SCZ	66	0.55	24.2	8.8	26.4	20.7	66	0.55	24.5
Li2021	SCZ	136	0.45	24.1	14.7	21.7	20.2	146	0.44	24.2
Qiu2021	BD	20	0.75	22.8	/	/	/	40	0.63	21.2
Solis2017 (auditory hallucinations)	SCZ	19	0.32	40.05	193	17.89	21.47	20	/	37.75
Solis2017 (without auditory hallucinations)	SCZ	14	0.43	36.43	96	11.43	14.36	20	/	37.75
Wu2019	SCZ	32	0.5	30.94	8.91	20	20.59	32	0.34	31.37
Yu2014	SCZ	69		31.7	85.2	12.1	13.4	62		29.9

### Main analysis results

Several areas exhibited statistically significant differences in ALFF values between the Schizophrenia and control groups prior to the application of FWE correction (**Table 2A**). Patients with schizophrenia displayed reduced ALFF values in the midcingulate cortex (z = -2.09, cluster size = 444 voxels), the left superior frontal gyrus (z = -2.05, cluster size = 343 voxels) and the postcentral gyrus (z = -2.07, cluster size = 110 voxels) as compared to the controls, while ALFF was increased in the right striatum (z = 2.89, cluster size = 537 voxels). No result survived FWE correction. No significant differences were detected regarding fALFF values when comparing schizophrenic patients with controls.

**Table 2A:** Schizophrenia, ALFF.

MNI coordinate	SDM-Z	P	Voxels	Description
(x,y,z)				
24,2,4	2.889	0.002	537	Right striatum
4,6,42	-2.093	0.018	444	Right median cingulate/paracingulate gyri, BA 24
-2,62,-6	-2.049	0.02	343	Left superior frontal gyrus, medial orbital, BA 10
50,-4,28	-2.074	0.019	110	Right postcentral gyrus, BA 4

As less than five studies were found for Bipolar and Schizoaffective Disorder, no separate meta-analysis was attempted for these specific diagnoses.

Regarding the spectrum of psychosis, several areas showed statistically significant differences between patients and controls both for ALFF and fALFF (**Tables 2B**, **C**). Areas with decreased ALFF in psychotic patients as compared to healthy controls included the right midcingulate cortex (z = -2.16, cluster size = 621 voxels), the left superior frontal gyrus (z = -2.01, cluster size = 95 voxels) and the right postcentral gyrus (z = -2.32, cluster size = 654 voxels). Moreover, patients with psychosis showed increased ALFF in the right striatum (z = 2.41, cluster size = 537 voxels). These results showed an almost complete overlap with the previous analysis on patients with schizophrenia, indicating a higher contribution to the final results by this sample. Finally, significant differences in fALFF were detected in the following areas: left insula (z = 2.66, cluster size = 2391 voxels), right precentral gyrus (z = -2.36, cluster size = 253 voxels), right striatum (z = 3.09, cluster size = 313 voxels), left superior frontal gyrus (medial orbital, z = -2.30, cluster size = 544 voxels), right inferior occipital gyrus (z = -2.05, cluster size = 328 voxels). No result survived FWE correction.

**Table 2B:** Psychosis, ALFF.

MNI coordinate	SDM-Z	P	Voxels	Description
(x,y,z)				
18,6,-2	2.415	0.008	537	Right striatum
50,-4,28	-2.323	0.01	654	Right postcentral gyrus, BA 4
6,2,40	-2.162	0.015	621	Right median cingulate/paracingulate gyri, BA 24
-4,62,-4	-2.006	0.022	95	Left superior frontal gyrus, medial orbital, BA 11

**Table 2C:** Psychosis, fALFF.

MNI coordinate	SDM-Z	P	Voxels	Description
(x,y,z)				
-42,-4,-2	2.656	0.004	2391	Left insula, BA 48
22,6,4	3.09	0.001	313	Right striatum
-4,46,-10	-2.295	0.011	544	Left superior frontal gyrus, medial orbital, BA 11
34,-92,-10	-2.046	0.02	328	Right inferior occipital gyrus, BA 18
44,-10,52	-2.235	0.013	253	Right precentral gyrus, BA 6

### Linear regression analysis

Regression analysis showed several significant areas of correlation between variables of interest (sex, age, duration of illness and mean PANSS score) and spatiotemporal dimensions of resting state fMRI. Among Schizophrenia only, as well as the overall spectrum of psychosis, ALFF in the right anterior thalamus was significantly decreased (z = -3.84, cluster size = 314 voxels) and negatively correlated with age (higher age, lower ALFF; β = -3.83). Further details are displayed in **Supplementary Table 3**. Heterogeneity (I^2^) in this region was equal to 8.43%, which can be classified as a low risk of empirical bias (33).

The fALFF regression in the sample of patients with Schizophrenia did not show any significant result. Conversely, the analysis of fALFF in the overall psychosis spectrum sample highlighted different areas of significant correlations with variables, with the following β correlation coefficients: right supplementary motor area (z = -2.89, cluster size = 1401 voxels, sex = -0.21, age = 0.21, duration of illness = -0.17, PANSS = 0.14), right precuneus (z = -2.89, cluster size = 938 voxels, sex = -0.19, age = 0.21, duration of illness = -0.16, PANSS = 0.14), right middle frontal gyrus (z = -2.84, cluster size = 444 voxels, sex = -0.17, age = 0.21, duration of illness = -0.17, PANSS = 0.14). Heterogeneity between studies in these areas was high (right supplementary motor area 58.56%, right precuneus 59.26% and right middle frontal gyrus 62.51%). Please see **Supplementary Table 3** for further details.

### Control analysis and FWE correction

None of the results obtained survived FWE correction in the subgroup of patients with Schizophrenia or in the clinical spectrum of patients with psychosis, both for ALFF and fALFF meta-analyses. In order to test whether this effect should be attributed to external variables, separate analyses accounting for the role of covariates of interest (sex, age, duration of illness, PANSS scores) were performed before FWE correction. No significant differences were retained after FWE. Further details are listed in **Supplementary Table 3.**

### Publication bias

Significant publication bias (p = 0.011, z = -2.55) was detected only for ALFF, in particular for the sample of patients with Schizophrenia. This bias was observed for ALFF values in the right anterior thalamus. Further details are available in **Supplementary Table 3.**

## Discussion

Our meta-analysis reveals an interesting trend suggesting reduced ALFF in the midcingulate cortex, left superior frontal gyrus and postcentral gyrus among patients with psychosis-spectrum disorders. On the contrary, we observed increased ALFF in the right striatum in patients with psychosis. Moreover, patients with psychosis-spectrum disorders exhibited a trend of reduced fALFF in the right precentral gyrus and right inferior occipital gyrus, while fALFF was increased in patients compared to controls in the left insula and in the right striatum.

### ALFF and fALFF results

When results were not corrected for multiple comparisons, a trend was observed for altered ALFF and fALFF among regions previously observed as divergent in schizophrenia or bipolar disorder within structural or functional neuroimaging studies. In fact, cingulate gyrus, which was previously found to be smaller on average in schizophrenia as compared to controls (55), and exerting a divergent pattern of connectivity with other cortical structures (56, 57), was here found as also characterized by lower ALFF. The cingulate gyrus has been implicated in nociception, threat detection, fear evoked behaviors, and avoidant mechanisms of response (58). For these reasons, its altered state during psychosis could be reflective of the anguish experienced by patients during psychosis, as well as the potential engagement in social avoidance and withdrawal during acute symptomatic episodes.

The frontal gyrus, also previously showing evidence of alterations in schizophrenia and bipolar disorder, has previously been interpreted as underlying cognitive impairments during acute episodes (59). Moreover, together with the postcentral gyrus, which was here observed as also exhibiting lower ALFF in the psychosis-spectrum, it has been implicated in verbal hallucinations (60). Alterations observed in fALFF also pertained to regions implicated in hallucinations and affective disturbances during acute psychosis, namely the striatum, the precentral gyrus and the insula (61). Additionally, the occipital gyrus has shown preliminary evidence of cortical thickness reduction in high-risk states for psychosis (62), while also being correlated with negative symptom severity in first-episode psychosis (63).

The complex interplay between hallucination status, affective domain alterations and symptomatic presentation in psychosis is yet to be fully elucidated. However, neuroimaging markers could then help to develop future early detection applications, possibly exploring the potential for vulnerability detection in high-risk populations.

In summary, these findings provide preliminary evidence for potential transdiagnostic alterations in brain activity in specific regions associated with psychosis. However, none of these findings in our meta analysis survived correction for multiple comparisons. This is noteworthy, especially when considering that none of the previously published meta-analyses on this topic corrected their results for multiple comparisons.

### Methodological remarks

Indeed, several findings from these previous studies were replicated as trending findings in the current meta-analysis, while others are divergent when compared to previous literature. For instance, Qiu et al. (64) described an increased spontaneous brain activity in the right middle frontal gyrus and in the left superior frontal gyrus in schizophrenia patients as compared to controls. Our meta-analysis suggested the findings of a potential difference in these regions, but in the opposite direction (patients with psychosis and schizophrenia having decreased ALFF and fALFF as compared to controls). This divergence in results could be due to the fact that Qiu et al. merged results from ALFF and fALFF studies. By contrast, Gong et al’s findings (9) of decreased ALFF in the postcentral gyrus in patients with schizophrenia were spotted by the present analysis. However, while Gong et al. found a significant difference bilaterally, the current synthesis only detected a trend-like difference in the right postcentral gyrus.

Notably, the previous meta-analytical approaches based on non-uniform methodologies may have observed statistically inflated results. While ALFF and fALFF might appear similar in their terminology, they reflect two separate physiological mechanisms: the first related to the power and amplitude of oscillations in the selected range, the second to the ratio of power for frequency components (65). In other words, while ALFF can be conceptualized as a measure of variation and amplitude in the frequency-domain, fALFF - as commonly employed - better reflects the relative dominance given by low-frequencies. For these reasons, previous meta-analytic works may have not correctly accounted for the divergent neurophysiological processes these two different measures might represent (66). Similarly, collating different measures such as regional homogeneity, ALFF, fALFF and cerebral blood flow in resting state fMRI might not have correctly represented the postulated underlying pathophysiological processes they reflect (67), and thus have contributed to the observed differences between synthetic evidence in these meta-analyses.

Moreover, while individual characteristics could also determine a heterogeneity in findings, irrespective of psychopathological dimensions and specific diagnoses, the current work shows that these effects do not pertain to areas individually interested by previous publications on the topic. In other words, significant results in single papers, or in the current meta-analysis before correction for multiple comparisons, do not seem to be significantly correlated with sex, age or the severity of positive or negative psychotic symptoms. Additionally, high heterogeneity was detected among fALFF studies (but not among ALFF studies). It could thus be hypothesized that other sources of heterogeneity might need to be explored. The pharmacological status of individuals in included studies was seldom reported, which did not allow for a meta-regression analysis on the effect of antipsychotic drugs on intrinsic brain activity (67). Similarly, no estimate of the role of smoking status could be assessed due to insufficient information, while being a potential confounder on cognitive and clinical correlates of Schizophrenia (68), Bipolar disorder (69), and a potential confounder for the BOLD response in fMRI (13). No estimate could also be derived for the role of motion (here measured by mean FrameWise Displacement per run), as insufficient data on this measure was present. Future research on ALFF and fALFF along the psychotic spectrum might then benefit from a standardization over collected clinical and experimental measurements, as well as from a better definition of methodological techniques employed.

The presence of publication bias in the current results could potentially explain the differences in heterogeneity between brain areas (70). This observation replicates previous evidence on the high heterogeneity among neuroimaging studies (16), further emphasizing the importance of controlling for the role of individual confounders. Since the right anterior thalamus displayed high publication bias, future studies might refrain from employing seed-based designs for spatiotemporal dimensions exploration in this population, as informed by the current literature on the topic.

### Clinical and research implications

The present meta-analysis did not show evidence for a potential difference in spatiotemporal dimensions of resting-state brain dynamics, as assessed by fMRI (ALFF/fALFF), between psychosis spectrum patients and healthy control. Nonetheless, the lack of statistically significant evidence at the present moment should not be interpreted as proof of no difference between groups, nor of no continuum between psychosis spectrum disorders, as the methodological quality and quantity of studies encompassing these constructs may currently hinder a generalization of results. Despite the lack of definite findings, these results suggest that clinicians could complement standard practice with translational and transdiagnostic investigations, as a full diagnosis of either Schizophrenia or Bipolar Disorder may prove challenging in the acute setting (71), and research on ALFF/fALFF may eventually lead to the identification of a biomarker for psychosis. For these reasons, a multidisciplinary assessment may be crucial in understanding future improvement in the diagnosis and prognosis of Schizophrenia and Bipolar Disorders, through the development of novel neuroimaging markers of disease. These neuroimaging markers could facilitate drug discovery, in the perspective of personalized medicine approaches.

Finally, the methodological considerations offered in the present study could be generalized beyond ALFF and fALFF. For example, a recent meta-analysis (72) on functional connectivity comparing patients with psychosis and healthy control found associations with several areas and the individual clinical status, but such results did not survive FDR correction. For these reasons, the current work offers evidence in support of a higher caution in the interpretation of single studies, or previous meta-analyses, updating the current theoretical frameworks for both Schizophrenia and Bipolar Disorder.

As a future research perspective, it could be relevant to cross-validate our findings by employing different techniques or metrics for resting-state fMRI analysis. Additionally, it is imperative to explore the effects of potential confounding variables that are currently not adjusted for in the ALFF literature. For example, patients with psychosis are at a heightened risk of metabolic syndrome (73), which holds significant clinical and research implications. Speculatively, these conditions could impact neuroimaging markers, potentially through the mediating role of inflammation. Therefore, future studies should investigate the influence of such variables to gain a more comprehensive understanding of the neurobiological underpinnings of psychosis.

### Limitations

Although all corresponding authors were contacted by email, no tridimensional statistical map was retrieved in order to perform image-based meta-analytical methods. Therefore, results reported in the included studies were meta-analytically analyzed by reported peak-activation given their coordinate and T-value. No study was retrieved evaluating ALFF/fALFF in individuals suffering from schizoaffective disorder; future studies evaluating this population would thus provide needed insight on the matter. Moreover, as data on psychotherapy treatment (in particular, if patients were undergoing psychotherapy interventions, the type of intervention adopted, and whether individuals underwent psychotherapy in the past) was sparse, the impact of psychological interventions was not accounted for in the current analysis. This perspective may be a relevant area of expansion for future research on ALFF/fALFF in Schizophrenia or Bipolar Disorder. Finally, as the field is constantly evolving, future methods and softwares could be employed.

## Conclusions

The current meta-analysis did not replicate previous studies on the topic. This result might be influenced by both methodological and statistical considerations. In fact, previous works did not account for significance inflation, although neuroimaging studies are known to be prone to Type I errors due to multiple comparisons. Moreover, ALFF and fALFF were previously collated, irrespective of the underlying neurophysiological processes they might represent. For these reasons, caution is warranted in the interpretation of previous results, and future empirical studies might seek higher replicability standardizing pre-processing (e.g., bandpass filters) or analytical procedures.

## Data availability statement

The original contributions presented in the study are included in the article/**Supplementary Materia**l. Further inquiries can be directed to the corresponding author.

## Author contributions

GM: Writing – original draft, Writing – review & editing. LT: Writing – original draft, Writing – review & editing. LFS: Conceptualization, Writing – original draft, Writing – review & editing, Data curation, Investigation, Methodology, Visualization. FD: Writing – original draft, Writing – review & editing. CP: Writing – original draft, Writing – review & editing. DV: Writing – original draft, Writing – review & editing. GC: Writing – original draft, Writing – review & editing. VR: Writing – original draft, Writing – review & editing.
