# Supplementary material for: Transdiagnostic markers across the psychosis continuum: a systematic review and meta-analysis of resting state fMRI studies
Source: Front Psychiatry. 2024 Jun 4;15:1378439. doi: 10.3389/fpsyt.2024.1378439 (PMC11184053; doi:10.3389/fpsyt.2024.1378439)
Supplement: Supplementary file 1 [file DataSheet_1.zip › Supplementary Figures.DOCX]

**Supplementary Figure S1 - Schizophrenia, ALFF, Grey Matter-Masked**


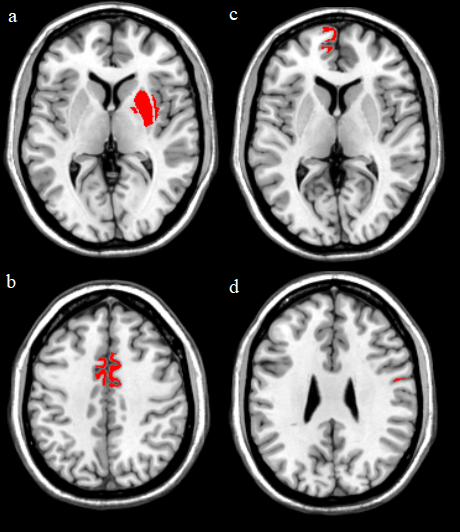


| ***Legend*** | ***MNI coordinate***  ***(x,y,z)*** | **Voxels** | **Description** |
| --- | --- | --- | --- |
| A | 24,2,4 | 537 | Right striatum |
| B | 4,6,42 | 444 | Right median cingulate / paracingulate gyri, BA 24 |
| C | -2,62,-6 | 343 | Left superior frontal gyrus, medial orbital, BA 10 |
| D | 50,-4,28 | 110 | Right postcentral gyrus, BA 4 |

#

**Supplementary Figure S2 - Psychosis, ALFF, Grey Matter-Masked**


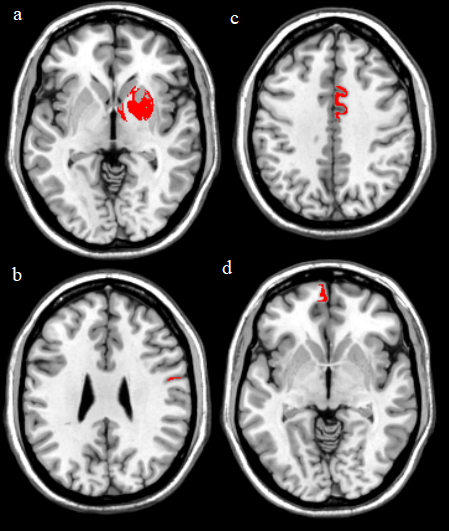


| ***Legend*** | ***MNI coordinate***  ***(x,y,z)*** | **Voxels** | **Description** |
| --- | --- | --- | --- |
| A | 18,6,-2 | 537 | Right striatum |
| B | 50,-4,28 | 654 | Right postcentral gyrus, BA 4 |
| C | 6,2,40 | 621 | Right median cingulate / paracingulate gyri, BA 24 |
| D | -4,62,-4 | 95 | Left superior frontal gyrus, medial orbital, BA 11 |

#

**Supplementary Figure S3- Psychosis, fALFF, Grey Matter-Masked**


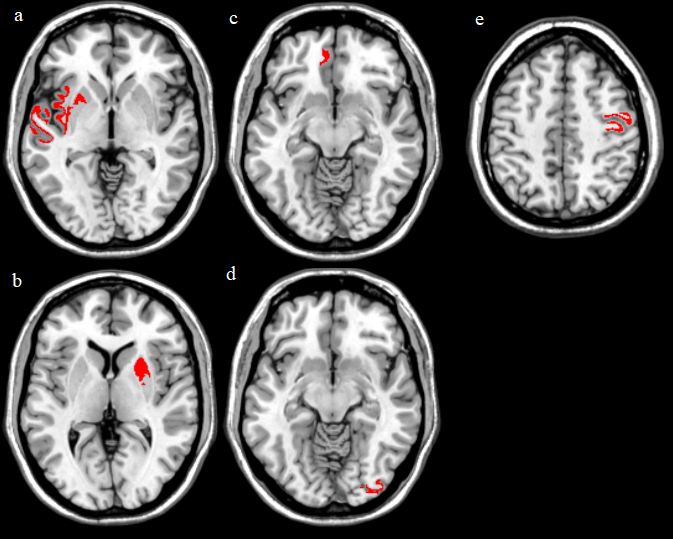


| ***Legend*** | ***MNI coordinate***  ***(x,y,z)*** | **Voxels** | **Description** |
| --- | --- | --- | --- |
| A | -42,-4,-2 | 2391 | Left insula, BA 48 |
| B | 22,6,4 | 313 | Right striatum |
| C | -4,46,-10 | 544 | Left superior frontal gyrus, medial orbital, BA 11 |
| D  E | 34,-92,-10  44,-10,52 | 328  253 | Right inferior occipital gyrus, BA 18  Right precentral gyrus, BA 6 |

# 
