# Supplementary material for: Transdiagnostic markers across the psychosis continuum: a systematic review and meta-analysis of resting state fMRI studies
Source: Front Psychiatry. 2024 Jun 4;15:1378439. doi: 10.3389/fpsyt.2024.1378439 (PMC11184053; doi:10.3389/fpsyt.2024.1378439)
Supplement: Supplementary file 1 [file DataSheet_1.zip › Supplementary Table 1.DOCX]

**Supplementary Table 1.** Search strategy according to the Population, Intervention, Comparison, Outcomes and Study Design (PICOS) model

| Parameter | Inclusion criteria | Exclusion criteria |
| --- | --- | --- |
| Population | At least some adult patients with a diagnosis of bipolar disorder (BD), schizophrenia (SCZ),or schizoaffective disorder (SAD), as assessed by the DSM, ICD. | - Absence of DSM or ICD diagnosis.  - No sub-analysis distinguishing BD, SCZ, SAD patients from other psychiatric patients or subjects  - Inclusion of non-adult (>=18years) subjects  - overlapping population with more recent paper |
| Interventions | - Amplitude of low frequency fluctuations (aLFF) Functional MRI (fMRI) measurements | - Other magnetic resonance techniques (e.g. magnetic resonance spectroscopy, MRS, or diffusion-tract imaging, DTI)  - Other fMRI techniques (e.g. dynamic aLFF) and no aLFF |
| Comparison | -Comparison between patients and healthy control subjects or other patients with different diagnoses (also subjects with a non-psychiatric illness).  -Comparisons between BD, SCZ, SAD patients (e.g. between patients in the same or different disease stages) | NA |
| Outcomes | NA | NA |
| Study design model | Observational (cross-sectional, retrospective or prospective) studies, and randomized controlled trials (RCT). | Case studies, case series, methods, conference presentations, and reviews or metanalysis. |
